# STI service delivery in British Columbia, Canada; providers' views of their services to youth

**DOI:** 10.1186/1472-6963-12-240

**Published:** 2012-08-06

**Authors:** Cindy L Masaro, Joy Johnson, Cathy Chabot, Jean Shoveller

**Affiliations:** 1 RN, MSN – Doctoral Candidate, University of British Columbia (UBC) Faculty of Applied Science/Nursing, 302 – 6190 Agronomy Road, Vancouver, V6T 1Z3, Canada; 2 PhD, RN, FCAHS– Professor, School of Nursing, UBC and Scientific Director, Canadian Institute of Health Research, Institute of Gender and Health, 302 – 6190 Agronomy Road, Vancouver, V6T 1Z3, Canada; 3 MA – Research Manager, School of Population and Public Health, UBC, 2206 East Mall, Vancouver, V6T 1Z3, Canada; 4 PhD – Professor, School of Population and Public Health, UBC, 2206 East Mall, Vancouver, V6T 1Z3, Canada

## Abstract

**Background:**

Little is known about service providers’ knowledge, attitudes, and experiences in relation to the assessment, diagnosis, and treatment of individuals seeking care for sexually transmitted infections (STIs), and how they influence the delivery of services. The purpose of this study was to explore the perceptions of STI care providers and the ways they approached their practice.

**Methods:**

We used a qualitative approach drawing on methods used in thematic analysis. Individual semi-structured in-depth interviews were conducted with 21 service providers delivering STI services in youth clinics, STI clinics, reproductive health clinics, and community public health units in British Columbia (BC), Canada.

**Results:**

Service providers’ descriptions of their activities and roles were shaped by a number of themes including specialization, scarcity, and maintaining the status quo. The analysis suggests that service providers perceive, at times, the delivery of STI care to be inefficient and inadequate.

**Conclusion:**

Findings from this study identify deficits in the delivery of STI services in BC. To understand these deficits, more research is needed to examine the larger health care structure within which service providers work, and how this structure not only informs and influences the delivery of services, but also how particular structural barriers impinge on and/or restrict practice.

## Background

The diagnosed incidence of sexually transmitted infections (STIs) continues to increase in Canada [1]. In the province of British Columbia (BC), the number of people testing positive for an STI has more than doubled in the past decade [2]. For Chlamydia, the most commonly reported STI in BC, the rate of diagnosed infection increased from 122.4 per 100,000 in 1998 to 239.3 per 100,000 in 2008 (a 96% increase). Among men and women during this same period, the highest rates of diagnosed Chlamydia were among those 20 to 24 years of age. Women in this age group had a rate (1743.3 per 100,000) more than twice that of the men in this same age group (832.3 per 100,000) [2]. Although the incidence of diagnosed STIs show a steady increase over this time period, we must also acknowledge that changes in the availability of STI testing, as well as changes in the population of those testing may, in part, have contributed to the rise in diagnosed incidence of these infections. Despite the increase in STIs, however, is concern that diagnosed cases far underestimate the number of people who are truly infected, but who remain unaware because of the asymptomatic nature of many STIs. Undiagnosed and untreated STIs can evolve into serious sequelae, particularly for young women, including pelvic inflammatory disease, infertility, chronic pelvic pain, ectopic pregnancy, and cervical cancer [3]. In addition, evidence now indicates that STIs enhance the transmission of human immunodeficiency virus (HIV) [4].

Increasing the number of sexually active individuals who seek testing for STIs is an important public health priority. Early diagnosis and treatment are key elements in preventing the transmission of STIs and reducing their sequelae. Several studies have identified barriers that reduce STI testing among people at risk. Such barriers include concerns about confidentiality, perceived stigma related to STIs [5,6], structural features related to testing (e.g., clinic location, hours of operation, availability of appointments), attitudes of health care providers towards users, and providers’ lack of knowledge and limited counseling skills [7,8]. Encounters with health care professionals represent opportunities for STI education and counselling for those at risk, and offer the potential to increase the uptake of STI services through the provision of quality STI care. Research related to the provision of STI services has focused on the screening practices of primary care physicians revealing a gap between ideal and actual practice [9-11]. For example, many physicians do not routinely assess STI risk, offer STI testing, or provide safer sex counselling to patients [12,13]. Barriers to providing appropriate STI services include inadequate training and specialization in the area of STIs, a reluctance to obtain a full sexual history to assess sexual risk because of discomfort in discussing these topics with patients, and a lack of time to provide adequate education and counselling within the typical encounter [6,9,14].

STI clinics provide another option for those seeking STI services. Weston et al.’s [7] systematic review of patient satisfaction found that most studies reported high levels of satisfaction among those attending STI clinics. In the United Kingdom (UK), the growing number of patients using STI clinics has resulted in increased pressure on those providing STI services [15]. As a result, an emerging issue in the literature concerns the expanded role of nurses in the delivery of sexual health care. Over the past two decades, nurses have taken on more responsibilities for STI services; responsibility that has traditionally been assumed by physicians [16,17]. In Canada (Achen, personal communication), the United States (US), the UK, the Netherlands, and Australia, nurse-led STI clinics have been in operation to meet the rising demand for STI services and to reduce onward transmission of STIs [18,19]. Several studies have shown that nurses perform as well, or better, than physicians in the delivery of quality STI care and their care is often rated higher for overall patient satisfaction [20-23].

Despite the growth in service provision related to STIs, little is known about STI service providers’ *knowledge, attitudes, and experiences* in providing care and, more specifically, how these may influence their practices during the delivery of STI services to youth. Furthermore, we argue that service providers’ perspectives on STI testing are shaped by broader themes that inform their practices. The purpose of this study was to examine how STI service providers working in British Columbia, Canada, viewed their roles and the services they provided to clients seeking care for STIs and how this in turn informed and influenced the delivery of STI services to clients.

### Context of STI service delivery in BC

In 2006 and 2007, when we conducted our interviews, STI services in BC were provided in many sites, including physician offices, youth clinics, STI clinics, sexual reproductive health clinics,, hospitals, and community public health units. Although historically physicians have been the principal providers of STI care in BC, since the late 1980’s, nurses employed at some facilities (e.g., youth clinics; public health units) also have been offering STI services under a system of delegated medical function, including: sexual history taking, risk assessment, counseling and education, specimen collection, examinations, ordering of diagnostic tests, and diagnosing and prescribing treatments for some infections.

## Methods

### Setting and participants

This study builds on the work of Shoveller et al. who investigated how socio-cultural influences (e.g., gender, culture and place) and structural features (e.g., clinic hours, locations, institutional policies) affected youth’s experiences accessing STI services [24]. For this study, we used a qualitative approach that drew on 21 individual in-depth ethnographic interviews conducted in 2006 and 2007 with service providers (n = 21; 18 women; 3 men; 23–65 years of age) who worked with youth in various capacities, including five physicians, 14 nurses, one administrative assistant, and one youth worker. Half of the nurses interviewed had completed the five day BC Centre for Disease Control (BCCDC) STI training course. This course was the only specialized training available to health care providers delivering STI services in BC at that time. Nurses delivering STI services were required to practice under delegated medical function. Completion of this course signified that nurses were prepared to deliver STI care to clients and practice in this capacity.

Service providers from a variety of clinical settings were interviewed, including: clinics that offered education, counseling, and a full range of STI testing; others that provided education and counseling only; and other clinics that provided testing with limited counseling. Thirteen of the interview participants worked in clinics located in BC’s largest urban centre (Vancouver area, population > 720,000), three worked in clinics located in a mid-sized geographically isolated urban centre in Northern BC (population ~ 85,000), and five worked in a small rural community in Northern BC (population < 10,000). The semi-structured individual interviews were conducted by four qualitatively trained interviewers ranging in age from their mid-twenties to early thirties. Three of the interviewers were Euro-Canadian women and one was a Euro-Canadian man. A more detailed description of the study settings has been provided elsewhere [see 24].

The semi-structured interviews with these service providers included questions about their experiences in providing STI services to youth, as well as the policies and practice guidelines that informed their work with youth. We asked their perspectives on the type of STI services available to youth in their communities, their experiences in providing these services, and the socio-cultural and structural conditions influencing the uptake of such services by youth in their communities. We used an interview guide consisting of semi-structured questions that were constructed to be open-ended allowing participants the freedom to elaborate and respond to prompts to expand upon and/or clarify their views (see attached). The guide included questions about the types of services offered, providers’ interactions with clients, STI assessment procedures, clients’ post-test responses, confidentiality issues, record keeping, and their satisfaction with the services they offered to youth. Participants also completed a brief socio-demographic questionnaire to obtain data describing their age, education, professional background, and current employment. All interviews were conducted in private settings and lasted approximately 60 to 90 minutes. Ethical approval for this study was obtained from the University of British Columbia and University of Northern British Columbia Research Ethics Boards, as well as the health authorities that oversee clinics in their regions.

### Analytical approach

We used a thematic analysis (TA) approach to analyzing our data. TA is a method that involves identifying and analyzing patterns of meaning or themes within qualitative data [25-27]. At a basic level, TA assists researchers in organizing and describing unstructured text-based data. Once a basic structure is identified, TA aids researchers in restructuring the data to develop an explanatory framework according to salient themes or aspects of the research topic that is consistent with the text [25,27]. One of the criticisms of TA concerns the fact that there have been no clear guidelines developed on how to go about conducting this type of analysis [27]. To fill this gap, Braun and Clarke [25] developed a step-by-step guide for conducting a TA (see Braun and Clarke for details), which we used to guide our analysis. The first step in the process involved transcribing all interview tapes verbatim and then reading and re-reading the text from each transcript in an active way searching for recurrent patterns, themes, topics, or relationships, which were identified manually by writing ideas or notes on specific segments of text that were related to the phenomena of interest. The second step involved generating initial codes from the ideas and notes identified in step one. The codes generated were intended to capture important aspects of the phenomena under study, and to represent some level of patterned response or meaning in the data set. In step three, we examined the codes and considered which ones grouped together to form a broader theme. The next step then involved refinement and interpretation of the themes. In refining themes we were seeking to determine if there was a clustering of themes and examined whether they formed a coherent pattern that adequately captured the coded data. In the interpretation phase we examined how the themes fit together, what was interesting about them and why, and then considered the overall story these themes conveyed about the data. The first author was primarily responsible for developing the initial codes and themes. Selected excerpts of coded texts supporting each theme were passed on to the second author for independent review. Refinement and interpretation of the themes were discussed with the entire research team. In presenting the findings we use pseudonyms, and identify the type of clinic where the provider worked, in order to situate the data.

## Results

The service providers’ descriptions of their activities and roles in their clinics were shaped by three themes including specialization, scarcity, and maintaining the status quo. These thematic perspectives were reflected in the way they explained the complexity of their work, how they practiced, and the challenges they faced. While one theme seemed to dominate the participants’ discussions, many of the service providers shifted between themes in the context of the interview as they sought to situate their practice in different ways. As we interviewed only those working in the province of BC, we acknowledge that our findings are not generalizable and limited only to service providers delivering STI care in this province.

### Specialization

The theme of specialization was often invoked to set STI practice apart from other forms of health care practice. Given the sensitive subject matter and stigma attached to STI testing, service providers described their practice as a *specialty* requiring a particular set of skills and understanding that not all service providers possess. Those who did not possess these specialized skills were “weeded out” and not afforded the opportunity of working in these settings, as pointed out by Robert, a nurse working in a busy urban STI clinic:

“You can’t work in this program unless you’re cool with gays…unless you’re cool with substance use.... I don’t have to use heroin to work in this program, but I have to be non-judgmental. If you have a judgmental thing happening…you’re weeded out in the interview process.”

Providers taking up this theme described themselves as more knowledgeable and skilled at providing STI care than those who do not specialize in sexual health. Some providers, mainly nurses, positioned their practice in opposition to primary care physicians, whom they perceived as “generalists.” They described generalists as those having some knowledge, but not an “in-depth” knowledge of STIs (primarily because they do not focus exclusively on sexual health and STIs). It was suggested by some that physicians were deficient in the knowledge and skills needed to adequately assess and test for STIs, as illustrated by Beth, a nurse working in a youth wellness clinic, who recounted one of her experiences:

‘I mean I’ve had doctors at the walk-in clinic who have treated somebody who’s symptomatic for Gonorrhea with the wrong medication…the one guy it happened to, I think he went in four or five times, and he was treated each time, and on the fourth or fifth time, had a urine test.... In the end it turned out he hadn’t been adequately screened, hadn’t been adequately treated.”

Providers who invoked the specialization theme also emphasized their ability to “spend time” with youth compared to other providers. For the majority of STI providers, time spent with a client was an important factor that determined the quality of STI care. In general, service providers argued that the more time one has to establish an environment conducive to the client’s disclosure of sensitive information, the more comprehensive the assessment and the deeper the understanding of both the medical and psychosocial aspects of sexual health. Mary, a salaried STI physician working in a busy urban STI clinic specializing in STI care made the following comment:

‘We find people that have been to four or five different walk-in clinics and they finally come, you give them time, sit them down, get the proper history and you’ve sorted them out…I think that the medical system should provide the ability to spend time so you can actually listen to people and they would save a lot of money in the long run…”

Mary thought that health care delivery could be improved by eliminating fee-for-service billing (FFS). She explained that because physicians bill FFS, they must restrict the time they spend with each client in order to maintain sufficient throughput in their daily billing – making it difficult to perform a comprehensive assessment and/or counselling. Physicians in primary care, who bill FFS, were most often perceived as not having enough time with clients and were frequently described as providing ‘piecemeal’ STI services. Beth, a nurse working in a youth wellness clinic explained:

“*They are providing Pap tests, and in addition to that they are doing screening but they are not doing the pre-counselling, they are not doing the HIV, not doing Syphilis or Hepatitis, so they’re doing a piece, but they’re not doing STD testing.”*

This approach was said to be detrimental to the welfare of youth largely because vital aspects of sexual health are not addressed. Ultimately, it was maintained that this led to substandard care and inconvenience for youth (e.g., requiring multiple clinic visits and further testing to resolve STI issues). FFS was described as the root of these problems by those who did not bill for health services in this manner.

Another aspect of the specialization theme was the particular emphasis placed on the need for confidentiality. Confidentiality was a predominant topic among STI providers as they viewed the privacy needs of youth as being much different from those seeking services in other areas of health care. Some clinics, mainly those in the Vancouver area, had the ability to provide testing that did not require youth to present their provincial personal health number (PHN) or to show ID. This was often contrasted with medical clinics, including walk-in clinics that require a PHN in order to bill the provincial Medical Services Plan (MSP) for services rendered. Several providers maintained that producing a PHN is problematic for some youth (e.g., their parents may have possession of their PHN cards). Others maintained that youth are at higher risk of potential confidentiality breaches (as compared to adults) because some providers, mainly physicians, were perceived not to be aware of or as understanding of the confidentiality issues associated with STIs. Valerie, a nurse working in an urban youth clinic explained, *“There’s doctors who say things like ’If a kid comes in to me I have to tell their parents.”* Thus, STI “specialists” distinguished themselves from generalists by highlighting the privacy advantage they believe they offer to youth seeking STI services.

The theme of specialization was also used to emphasize how providers were able to overcome the inadequacies of current policies related to STI/HIV services/care in meeting the needs of youth seeking STI services. Importantly, this theme was used to justify how providers moved beyond the rules and policies in order to meet youths’ needs. Erica, a physician in Vancouver, discussed how providers in her clinic offered youth the opportunity to opt out of having their names entered into the electronic record keeping system, despite the tension that this created with administration. Others such as Beth, a nurse working in a youth clinic described circumventing policy if she felt it to be beneficial for the youth. For example, although provincial policy (at the time of this interview) was to swab for Gonorrhea, Beth requested a urine test instead:

“we’ve been telling doctors who have tested people [who receive a] positive [for a STI], ‘you know you may want to confirm with a swab if this person isn’t high risk’. But if they’re high risk, I’m not waiting for a swab to treat them because they could disappear and never come back and…it could be too serious.”

In some instances, the specialization theme appeared to override young people’s agency, as providers suggested they knew what was best for young people because they were specialists. Difficulties experienced in providing care were located with youth, rather than the approach taken. In particular, STI care problems were thought to stem directly from youths’ lack of responsibility and sense of entitlement, as highlighted by Dorothy, a nurse working in a student health clinic:

“They [the youth] have the sense of entitlement or a sense of ‘someone is just going to give me condoms.’ Whereas they don’t have the responsibility to sort of say ‘It's my job to go out to BUY them or get them any way I CAN, that’s my responsibility, and not for someone to put them under my door, or given them to me for free.’"

Ray, a nurse working in a STI clinic, indicated that many youth seemed unwilling to listen closely to messages about safe sex and that *"everything you say is virtually irrelevant.“* Given the alleged lack of responsibility, these providers indicated that it was within their scope of practice to employ extraordinary measures to ensure that youth receive additional follow-up care. Dorothy described using whatever measures were required to locate someone with an STI, and implied that many youth are not responsible enough to get their test results:

“But if I’m concerned I also have ways of getting information…because if it’s really risky I won’t let it bother me. If someone was a -- had a positive HIV result…I’ll use whatever devious means, ‘cause I’ve done that before with the STD clinic, as you can just find some way around.”

Ruth, a nurse working in a youth clinic, indicated that, in addition to sexual health issues, she also tries to help youth with other health issues. She stated that she is able to consolidate her workload to deal with issues such as poverty and mental illness and suggested she knows what is best for youth in relation to these issues: *“I also know who needs a little boost and who doesn’t along the way.“* When service providers override young people’s agency, they also appear to invoke a sense of “ownership” in relation to youth (e.g., the youth need to be encouraged to return to the clinic; and, particular tactics are used because the providers know what is best). Perhaps most importantly, the providers describe talking to youth in such a way that suggests they are taking on a parental role:

Ruth - “and I had a fit one night because I had a young woman, obviously needed emergency contraception. I said, ‘Under no circumstances do I want your mother pulling a wallet out to pay for something that you need and I consider completely private. So let’s you and I make a little deal that I’m presenting it to you as a gift and you can just quietly tell your mother nothing.‘"

In this scenario, Ruth does not mention whether or not the young woman had discussed the need for emergency contraception with her mother. It may have been likely that this young woman's mother knew why her daughter was at the clinic, especially considering that the mother was present at the clinic and willing to pay. Even when youth have made a decision, Ruth describes intervening because she believes she knows what is best:

"I mean if I do a pregnancy options counselling, and even though I know they've pretty well made their decision but it's a little bit of roaring ambivalence that flies in. I said, ‘Let's you and I make a deal. My role really does end here…but let's follow up in two weeks…and so they'll get a phone call from me in two weeks’ time.’"

Ruth went on to describe how she kept a detailed list of youth whom she perceived were in need of additional support and follow up, even though their cases had been resolved. Unless youth had specifically requested or agreed to be followed up, follow up in these situation may be uncalled-for and create more anxiety or confidentiality concerns, especially if unwarranted voicemail messages are left for health issues a young person thought were resolved. Efforts at maintaining unnecessary relationships with the youth and a sense of "mothering" were invoked when Ruth suggested that youth must be physically present to discuss partner notification, rather than discussing this over the phone. Discussing partner notification can be conducted over the phone and does not necessarily require youth to be physically present in the clinic setting. In her interview Ruth recounted telling some youth who required partner notification:

"’So why don't you come in tonight?’… I mean I've had people say, 'Well it's not a good time.' ‘Well, it's never going to be a good time and what's going to make it positive for you? How about we do it tomorrow?’"

### Scarcity

Some providers made reference to notions of scarcity as they emphasised what could *not* be achieved because of a lack of resources, frequently describing themselves as ‘overwhelmed’ and ‘frustrated’. These providers recognized that certain STI services were lacking, and that their practice could be improved by offering these services. The theme of scarcity was characterised by a recognition that resources were lacking in the system, and an expression of frustration that things should change but cannot, as Beth, a nurse in Northern BC explained: *"We have always been really full.... We're at full capacity and we just don't have the resources to increase, which we need to do."* In the end this theme emphasised that STI providers were *“doing the best that they could do.”*

One of the main scarcity issues described concerned a lack of human resources. Providers expressed frustration with not having enough staff to cover existing services, to expand these services, or to hire new personnel to meet the needs of youth. For example, Ruth, a youth clinic nurse , told us:

"we need more staff. We need more nurses. That would be lovely if we had one more nurse doing more delegation and that sense of flow…it would be incredible. That's a daydream."

The theme of scarcity was also used in discussing practitioners’ physical space. Several providers stated that their clinics did not have enough space, or that the clinic location and hours of operation were not suitable for meeting their clientele's needs. Some described not being able to offer services because of the lack of material supplies, as illustrated by Anne, a nurse in Northern BC:

"So when we went down to the STI training [offered by the BCCDC], I came back, I still couldn't do women, even though we had been shown it. We didn't have a set up, we didn't have any means of doing it, we didn't have any of the equipment, we didn't have any lights, nothing."

### Maintaining the status quo

In this third theme, STI providers, mainly nurses, recognized that there are inefficiencies within the system yet, for the most part, they appeared resigned to these inefficiencies. A key element of this theme is an expression of resignation and frustration over things that cannot be changed, as evidenced by Helen, a public health nurse , who spoke about the process for follow up on positive test results: *"…time-consuming, that's frustrating. And I don't know if that's fixable or not."* In their descriptions of their practices, providers reflected this theme only indirectly. No practitioner indicated that they were in favour of the status quo, but this theme was hinted at as the providers suggested that they follow the routines and policies of the clinics even when they led to questionable outcomes.

In many cases nurse providers were qualified and competent to carry out several of the same functions as physicians. Clare, a youth clinic nurse explained:

"All of us that work here have a degree in nursing and then we've all taken extra courses at BCCDC, the STI clinic, that's one of the prerequisites."

However, at some clinics these nursing activities were restricted and not supported by administration or the physicians working there. Interestingly, the nurses at these clinics did not appear to dispute policies or guidelines that unnecessarily restricted their practice, perhaps in part because they felt unsure about their nursing responsibilities in relation to STI care, and what they were "allowed" to do and what they were "not allowed" to do. For example, Anne, a public health nurse discussed how she was unaware what nurses were permitted to do in terms of STI testing, diagnosis, and treatment until she heard information from another nurse provider:

"And so it's one of those…what you don't know, you sometimes don't question."

Others could not envision operating a STI clinic without a physician present for consultations: *"I couldn't imagine sort of constantly having to operate that way, because some clinics do, you know, it's strictly nurse-run, but it is limiting in terms of how much you could provide and your sense of what it means to have a touchstone."* (Ruth, a sexual and reproductive health clinic nurse).

Many nurses who were qualified to perform specific functions (e.g., Pap tests and STI tests) were ‘tasked’ with these responsibilities during busy time periods in clinics, but only completed these procedures when the clinic physician was too busy and there was a back up of clients in the waiting room. In some clinics, there appeared to be incorrect delineation of tasks between physician and nurse, which in the following case resulted in a lengthy delay in following up with youth who have tested positive for a STI at a physician's office:

*"If Dr. X gets a gonorrhoea, we have--sometimes a month delay before we're allowed to try and contact that person…because we have to get permission from him to contact them. … If it was up to us, we could probably do it faster but that might interfere with the doctor-client relationships, [which] we're not allowed to, so…"* (Helen, public health nurse)

Despite being qualified to provide STI care and being aware of the problems and inefficiencies within the system, providers invoked the status quo and did not discuss how their own skills or qualifications could be part of the solution. Anne, a public health nurse , began to understand this only after a new nurse, who questioned the status quo, began working at her clinic:

Julie is just very good at pushing it until it happens. You know, it made no sense that we couldn't give it [herpes, trichomoniasis, bacterial vaginosis treatment]. They give it down at BCCDC. And then we found out that they were giving it in [name of city] but it still took a lot--it's that whole getting through the paperwork and the hierarchy to be allowed to actually do it. So we are now and she's phenomenal, that's how we get so much going."

## Discussion

Perceptions about matters such as STI practice are shaped by institutional and social structures (e.g., formal and informal policies, practices, procedures, power dynamics) that enforce particular ways of thinking and acting. In this study we sought to understand how certain language and social processes around STI service delivery influenced the ways in which service providers thought and talked about particular aspects of their practice, and how this influenced the care they provided to youth. Several features of the themes identified in this study reflect service providers’ perceptions that the delivery of STI care is at times inefficient and inadequate. Although these themes have been identified in relation to service providers' experiences delivering care to youth, similar themes have been identified among those providing STI services to adults [28]. Thus, our findings may be applicable to STI service delivery in BC in general. While it is tempting to “blame” the service providers for these deficiencies, we must first look to the larger health care structure within which the service providers work, and give consideration to how this structure informs and influences practice and thus service delivery, as well as the structural barriers that limit practice. In the following discussion we highlight key structural factors that shape STI testing.

The theme of specialization highlights the need felt by many STI service providers to develop a specialized body of knowledge in relation to the provision of STI services. This specialized knowledge was required because of the sensitivity associated with having an STI, the importance of spending time building rapport and obtaining an accurate sexual health history. Those whose discussion reflected this theme perceived clients of STI services to have needs that were quite different from those accessing other primary care services. The need for a specialized approach may stem, to some extent, from society’s negative social judgment, which is often stigmatizing and discriminating, toward those who acquire STIs. Individuals receiving a STI diagnosis are often viewed as dirty and promiscuous [29] and report experiencing shame, discomfort, anxiety, isolation, and rejection [30,31]. This results in the need for providers to break down barriers to open communication. The need for service providers to address these issues was seen in sharp contrast to traditional STI services that focus on the diagnosis and treatment of the disease rather than include the socio-cultural context that influences and limits people's decision making in relation to their sexual health.

A key feature of the specialization theme was an emphasis on the need to spend more time with clients. It was argued that this enabled service providers' opportunities to understand and deal with the multifaceted psycho-social and socio-cultural aspects of their clients' sexual health, which they perceived as necessary for providing a higher standard of care. Notably, this theme often involved contrasting the service providers remunerated by FFS with those on a fixed salary. For example, service providers billing FFS were deemed to provide substandard care because considerably less time was spent consulting with the client about the socio-cultural and structural conditions that constrain sexual health and well-being; the focus instead was on the medical-curative aspects. Indeed, research demonstrates that service providers who are not paid on a FFS basis spend more time providing patients with direct patient care (e.g., longer consultations, follow up) [32]. According to Devlin and Samra, the FFS form of remuneration is a formidable tool for influencing physician behaviour. For example, FFS physicians supplement their income by increasing the number of clients they see rather than by increasing the quality of the services they provide. Decisions regarding care are often determined by the desire to generate income rather than the desire to serve the needs of the client [33]. The FFS model rewards rapid, technical procedures over the delivery of holistic care [3].

Also apparent in the specialization theme is the existing tension between non-physician and physician STI service providers, which may in part, stem from a lack of recognition of the full role that non-physician providers (e.g., nurses) are able to play in relation to STI service delivery. Indeed, expectations about the role STI service providers play in the delivery of STI services shows an underlying “tug-of-war” for power and ownership between physicians and non-physician service providers in their attempt to shape the system. At the time of our interviews, non-physician STI service providers were not formally recognized as specialists in this area. The specialization theme was reinforced by the perceived need for non-physicians to establish credibility and decrease dependence on physicians. Jones [34] suggests that unclear role expectations underlie some healthcare providers’ negative attitudes toward expanded practice roles. Turf battles and concerns about whether service providers are practicing beyond their scope of practice arise, especially when there is an overlapping of roles [35]. Clients can also express concern. Rashid’s [36] findings show that when roles formerly undertaken by physicians were carried out by nurses practicing in an expanded role, clients were more likely to express concern about the nurses’ level of training and competency, particularly in relation to diagnostic testing and treatment.

Although some service providers felt the need to ‘create a niche’ for themselves in the provision of STI services, what also needs to be considered is how specialization can create obstacles for others to provide STI care. At the time of our interviews service providers could only attend the BCCDC STI training course if they were nominated by their employers, and had their time and expenses paid. This limited the number of practitioners who were specially trained to provide STI care. Another unintended obstacle created by specialization is the tendency to disregard the knowledge and experience of youth. Service providers whose comments suggested a patronizing attitude may have been expressing their own disciplinary power, as knowledge and specialization are recognized tools for exercising power over others [37].

Notions of scarcity influence much of health care policy [38]. As such, it is not surprising that it was used to position STI testing. Federal and provincial government budgets have become increasingly dominated by the high costs of health care. Providers readily described how they lacked appropriate human and physical resources, and identified these as limitations in the provision of care, but there was minimal discussion about how these issues might be addressed. The knowledge gained through training programs is a necessary factor for initiating change, but inadequate resources (e.g., support staff, technology, infrastructure) place service providers at an extreme disadvantage when trying to practice in an expanded role and have been frequently reported in the literature [32]. As evidenced in some settings, structural conditions (e.g., policies, procedures, practices) were such that implementing aspects of an expanded STI service role was not possible. Coordination and expansion of STI services pose major challenges for provincial governments as they attempt to reconcile increasing demand with budgetary constraints. This has resulted in much emphasis being placed on improving service delivery using existing resources [32].

In recent years there has been an increasing demand on non-physician service providers to deliver quality STI care [22]. The findings, however, reveal a lack of clarity surrounding the role of non-physician service providers, and a lack of support for STI education, training, and organizational resources for those delivering this care and practicing in an expanded role. In some areas, these structural constraints have resulted in STI services that are fragmented with little or no coordination. According to Kumpers, van Raak, Hardy, and Mur [39], this causes waste and undersupply on the system’s side and undersupply and neglect for those using the services. In a systematic review by Jones [34], lack of role clarity was identified as one of the most important factors limiting the implementation of an expanded practice role. Lack of role clarity contributes to confusion among other healthcare service providers about the activities performed by those practicing in an expanded capacity [34]. This may limit the acceptance of these roles by other providers, hinder innovation, and perpetuate the status quo. Providing well-defined goals and expectations for expanded practice roles would provide greater recognition for those practicing in these roles [35].

When people become worn down by the system, they eventually give in and accept the status quo [2,40]. In this way, the system limits service providers in thinking about and initiating change. Some service providers may have disengaged from attempting to make changes due to frustration experienced from confrontational situations with physicians or administrators, especially in situations where they perceive themselves to have limited power. Service providers in the current study frequently said they lacked the power to make structural changes and, as result, worried that they were inadvertently reinforcing the existing system. Because of these constraints, some service providers resorted to employing strategies that offered innovations in practice because they circumvented existing policy and procedures. Subsequently, service providers’ creative energies were diverted to overcoming barriers rather than in advocating for change.

Limitations of this study should be acknowledged. First, the study focused on a particular context and the findings are not necessarily generalizable to other contexts. Second, we focused on the perspectives of care providers and this understanding could be bolstered by consideration of the actual day-to-day actions and practices, which will be the focus of a subsequent manuscript. Also, TA is interpretive and the themes that are described herein result from a specific analytical orientation; other themes may be revealed by other analytic strategies.

## Conclusion

The findings of this study provide valuable insights regarding perspectives that shape STI service delivery to youth. These findings call into question approaches that focus solely on educating individual practitioners and point to the need to also address important structural factors that limit STI service delivery.

## Competing interests

The authors declare that they have no competing interests.

## Authors’ contributions

CLM conducted the data analysis and drafted the manuscript. JJ reviewed and provided feedback on the data analysis and the manuscript. CC and JS also provided feedback both on the data analysis and manuscript. JS conceived of the original study. All authors read and approved the final manuscript.

## Pre-publication history

The pre-publication history for this paper can be accessed here:

http://www.biomedcentral.com/1472-6963/12/240/prepub
